# Supplementary material for: Knowledge gaps and acquisition about HPV and its vaccine among Brazilian medical students
Source: PLoS One. 2020 Mar 19;15(3):e0230058. doi: 10.1371/journal.pone.0230058 (PMC7082043; doi:10.1371/journal.pone.0230058)
Supplement: S1 Table — (PDF) [file pone.0230058.s001.pdf]

Questions	Sex		PR (CI 95%)*
	Male	Female	
1. Do you know what HPV is?	302 (96.8)	198 (96.1)	1.0 (0.9 : 1.0)
2. Is HPV a virus?	311 (99.7)	206 (100)	1.0 (1.0 : 1.0)
3. Is HPV a sexually transmitted disease?	297 (95.2)	195 (95.6)	1.0 (0.9 : 1.0)
4. Can HPV cause cervical cancer?	308 (98.7)	204 (99)	1.0 (1.0 : 1.0)
5. Can HPV cause changes in Pap smear	281 (90.3)	192 (93.2)	1.0 (0.9 : 1.0)
6. Is cervical cancer a leading cause of death in women?	284 (91.3)	184 (89.3)	1.0 (1.0 : 1.1)
7. Can smoking increase the risk of cervical cancer?	183 (58.8)	126 (61.2)	1.0 (0.8 : 1.1)
8. Does the HPV vaccine prevent cervical cancer?	214 (69.3)	162 (79.0)	0.9 (0.8 : 1.0)
9. Should the HPV vaccine be given before the first sexual intercourse?	225 (72.1)	156 (75.7)	0.9 (0.8 : 1.0)
10. Can the HPV vaccine be given to people who have had sex?	261 (83.6)	185 (89.8)	0.9 (0.9 : 1.0)
11. Can the HPV vaccine be harmful to your health?*	108 (34.7)	85 (41.3)	0.8 (0.7 : 1.0)
12. Can the HPV vaccine cause HPV infection?*	196 (63)	157 (76.2)	0.8 (0.7 : 0.9)
13. Is the HPV vaccine provided by the government?	254 (81.9)	178 (86.4)	0.9 (0.9 : 1.0)
15. Is the HPV vaccine part of the girls' immunization records?	168 (54)	143 (69.4)	0.8 (0.7 : 0.9)
16. Are 3 doses required for complete vaccination?	128 (41.2)	144 (71.6)	0.6 (0.5 : 0.7)
17. Does the HPV vaccine decrease the chance of having genital warts?	201 (64.8)	135 (67.2)	1.0 (0.8 : 1.1)
18. Does the HPV vaccine decrease the chance of having changes in the Pap smear test?	230 (74.4)	165 (82.5)	0.9 (0.8 : 1.0)
19. Do you think the HPV vaccine will stimulate the onset of sexual activity at an earlier age?*	280 (90.3)	193 (96.0)	0.9 (0.9 : 1.0)
20. Do you think that you still need to use a condom after HPV vaccination?	309 (100)	201 (100)	1.0
21. Do you think that you still need to have a Pap smear test after HPV vaccination?	308 (99.7)	200 (99.5)	1.0 (1.0 : 1.0)
22. Do you know anyone who has already received the HPV vaccine?	158 (50.8)	150 (74.6)	0.7 (0.5 : 0.8)

**23. Have you received the HPV vaccine yet?**	19 (6.1)	75 (37.3)	0.2 (0.1 : 0.3)
**24. Would you recommend the HPV vaccine for a child, friend, or relative?**	274 (88.1)	169 (84.5)	1.0 (1.0 : 1.1)
**28. Can patients living with HIV get the vaccine?**	115 (37.6)	65 (32.3)	1.2 (0.9 : 1.5)
**29. Am I confident to indicate HPV vaccination for patients?**	200 (65.4)	150 (74.6)	0.9 (0.8 : 1.0)
**30 Do I feel confident giving information about HPV to patients?**	168 (55.1)	119 (59.5)	0.9 (0.8 : 1.1)
**31 Can pregnant patients get the vaccine?**	17 (5.6)	14 (7.0)	0.8 (0.4 : 1.5)

***PR (CI 95%): Prevalence ratio (Confidence Interval of 95%) calculated by Poisson regression.**
