# Supplementary material for: Knowledge gaps and acquisition about HPV and its vaccine among Brazilian medical students
Source: PLoS One. 2020 Mar 19;15(3):e0230058. doi: 10.1371/journal.pone.0230058 (PMC7082043; doi:10.1371/journal.pone.0230058)
Supplement: Attachment — Submitted filename: Response to reviewers 19.02.2020.docx [file pone.0230058.s003.docx]

Dear Editor,

We are grateful for the considerations about our manuscript, "Knowledge Gaps and Acquisition About HPV and its Quadrivalent Vaccine Among Medical Students" (Manuscript ID: PONE-D-19-33466), to PLOS ONE.

This response letter contains a point-by-point reply to the peer reviewer’s comments, outlining the changes we have made.

**Reviewer #1**

**Comment 1: No comments**

**Reviewer #3**

**The topic addressed in the manuscript is important in terms of public health. It seems to me that the authors addressed properly all comments raised by the previous reviewers. The paper may be published and I have only some minor comments.**

**Comment 1:**

**“In the abstract, the Cronbach's alpha value of 0.74 should be reported in the results section.”**

**Answer to Comment 1:** We kindly appreciate the suggestion and made the necessary changes in the abstract:

“**Results:** To evaluate the internal consistency of the instrument applied, the Cronbach's alpha equation was used, obtaining the alpha value (α) = 0.74 for this population. This value attests that the consistency of the answers obtained with this questionnaire is considered substantial and acceptable.”

**Comment 2:**

**“In the results section the paragraph concerning the internal validity of the questionnaire should be at the beginning and not at the end of the section.”**

**Answer to Comment 2:** We kindly appreciate the suggestion and put the paragraph concerning the internal validity of the questionnaire at the beginning of the section Results.

**Comment 3:**

**“There is an error in the reporting of 95% CI of the PR concerning the year of study, both in the abstract and in the result section.”**

**Answer to Comment 3:** We kindly appreciate the correction and made the necessary changes in the abstract and in the results section:

**Abstract:** “Students in the first, second and third year of study had a 51% higher risk of a knowledge gap when compared to students in the final years of graduation [PR 1.51 (1.3:1.8); p <0.001].”

**Results:** “It is observed that there was a difference in students by grade with first, second and thrid year students having a 51% risk of having a knowledge gap than students in the final cycle [PR 1.51 (1.3:1.8)]”
